# Editorial: Exploiting the potential of native and modified legume proteins for the development of functional foods

**DOI:** 10.3389/fnut.2024.1361730

**Published:** 2024-01-30

**Authors:** Hafiz Rizwan Sharif, Mian Kamran Sharif, Husnain Raza

**Affiliations:** ^1^Department of Food Science and Technology, Faculty of Science and Technology, University of Central Punjab, Lahore, Pakistan; ^2^National Institute of Food Science and Technology, Faculty of Food, Nutrition and Home Sciences, University of Agriculture Faisalabad, Faisalabad, Pakistan; ^3^Department of Food Science, University of Copenhagen, Copenhagen, Denmark

**Keywords:** legume proteins, modified legume proteins, structural and functional properties, functional food, applications

Proteins are essential macronutrients necessary for energy, body growth, and functioning. Legumes are crucial crops and a significant food source after cereals. They provide energy and offer health benefits due to the presence of nutraceuticals with high biological potential. Protein energy malnutrition significantly impacts South-Asian and African populations. Legume proteins emerge as a viable and cost-effective alternative to animal proteins, and their use can help for the development of sustainable food systems to address food insecurity and malnutrition. The food industry has recently shown a great interest in using plants as substitutes for animal and dairy proteins in the development of various functional foods. In addition to their nutritional value, legume proteins possess physico-chemical properties that define the functional characteristics of food systems during processing, storage, and consumption. These properties include solubility, foaming, gelling, and emulsification (1). This research theme aims to highlight the importance of legume proteins and their modifications in the development of functional foods to address protein energy malnutrition. It also aims to create legume protein-based edible films that carry antimicrobial and antioxidant agents. Furthermore, it explores the effects of processing techniques on the structural and functional properties of legume proteins, as well as their applications in improving overall health.

Nowadays, sustainable use of resources is a focus of interest and need of the time as well. Producing plant protein requires fewer resources such as land, water, and inputs compared to producing the same amount of animal protein (2). Furthermore, consumer health and environmental considerations drive the development of plant-based proteins. This theme has been highlighted by Latif et al. who reviewed the importance of alfalfa as a leading herbaceous legume. The review outlined alfalfa's role and potential in agriculture, food, and feed. Alfalfa demonstrates environmental-friendly characteristics, tolerating different stress conditions and positively influencing the environment by controlling weeds, purifying water, controlling soil erosion, and fixing nitrogen. It also has the potential to be used as feed for dairy animals and provides a beneficial nutraceutical profile with positive effects on human health.

Singh et al. have discussed the importance of legumes for promoting a healthier lifestyle and addressing protein energy malnutrition. Due to their affordability and high protein content, micronutrients, and phytochemicals, legumes are beneficial for people with celiac disease, diabetes, and health-conscious individuals. Legumes can be utilized in various foods, including bakery products, pasta, snacks, beverages, infant formulas, and baby foods, to exploit their potential. However, soaking, boiling, roasting, microwave heating, or combinations of these methods can help reduce the anti-nutritional factors present in legumes before consumption (3).

Traditional nutrient determination methods are relatively expensive and time-consuming. Near Infrared Reflectance Spectroscopy (NIRS) combined with chemometrics offers non-invasive and time-saving techniques as alternatives to traditional evaluation methods. John et al. developed a model for the quick nutritional profiling of 20 underutilized legume cultivars. Various processing techniques are essential for improving the structural and functional properties of proteins and their applications in food (4). Mudgil et al. prepared chia and flax seed protein hydrolysates using various proteases to evaluate their *in-vitro* anti-diabetic, anti-obesity, and antioxidant properties. The functionality of the hydrolysates significantly improved as the degree of hydrolysis increased over time. Food packaging materials are crucial for preserving nutrients and maintaining their safety and freshness. Research has been carried out to create edible, smart, functional, and biodegradable packaging with improved antioxidant and antimicrobial properties. Abdullah et al. reviewed the impact of different plant and animal-based biopolymers, fabrication methods, and functionality for their potential as packaging materials. Biopolymer-based packaging films/materials are gaining attention and interest due to their natural, clean label features and their biodegradable, renewable, and eco-friendly status.

In Summary, the articles in this Research Topic highlight the importance of legume protein, their applications for the development of functional foods and healthier diets. Further, the right use of legume's potential could eventually lead toward sustainability, food security and mitigation of malnutrition.

## Author contributions

HS: Conceptualization, Investigation, Supervision, Writing—original draft, Project administration, Visualization, Writing—review & editing. MS: Investigation, Project administration, Supervision, Writing—review & editing, Writing—original draft. HR: Investigation, Project administration, Validation, Writing—review & editing, Writing—original draft.
